# Intravitreal Sustained Release of Dexamethasone from a Self-Healing Injectable Hydrogel: An In Vivo Safety and Release Study

**DOI:** 10.1021/acs.molpharmaceut.5c00872

**Published:** 2025-09-30

**Authors:** Ada Annala, Amir Sadeghi, Elisa Toropainen, Annika Valtari, Jooseppi Puranen, Jussi J. Paterno, Lea Pirskanen, Kati-Sisko Vellonen, Wim E. Hennink, Marika Ruponen, Tina Vermonden, Arto Urtti

**Affiliations:** † Division of Pharmaceutics, Utrecht Institute for Pharmaceutical Sciences, Faculty of Science, Utrecht University, Universiteitsweg 99, 3584 CG Utrecht, The Netherlands; ‡ School of Pharmacy, 163043 University of Eastern Finland, 70210 Kuopio, Finland; § Division of Pharmaceutical Biosciences, Faculty of Pharmacy, University of Helsinki, 00014 Helsinki, Finland

**Keywords:** dexamethasone, hydrogel, controlled release, intravitreal injection, pharmacokinetics, block copolymer

## Abstract

Corticosteroids, such as dexamethasone, are clinically used in intravitreal injections for the treatment of inflammatory and age-related ocular diseases; however, frequent injections can cause complications. To prolong the retention of dexamethasone in the eye after intravitreal administration, sustained-release drug delivery systems have previously been investigated. The aim of this study was to evaluate the in vivo release of dexamethasone from a self-healing thermosensitive hydrogel consisting of a thermosensitive ABA triblock copolymer and to investigate its safety after its injection into the eyes of rats and rabbits. The polymer building block for hydrogel preparation was synthesized by copolymerization of a methacrylated dexamethasone prodrug (mDEX) with *N*-isopropylacrylamide (NIPAM) and *N*-acryloxysuccinimide (NAS) through reversible addition–fragmentation chain transfer (RAFT) polymerization, using poly­(ethylene glycol) (PEG; 6 kDa) functionalized at both ends with a chain transfer agent (CTA). This yielded a thermosensitive triblock copolymer (p­(NIPAM-*co*-NAS-*co*-mDEX)-PEG-P­(NIPAM-*co*-NAS-*co*-mDEX) with a cloud point of 23 °C. Upon incubation of an aqueous solution of this polymer at 37 °C, thermogelation occurs. The resulting thermogel is chemically stabilized by cross-linking with cystamine, a compound with two amino groups that react with the succinimide functionalities present in the polymer chains. Intravitreal injections of a preformed fluorescently labeled hydrogel into rats were carried out, and hydrogel degradation and retinal health were followed using optical coherence tomography (OCT) and fundus imaging. The hydrogel started to degrade 2–3 weeks post injection, and it was cleared from the eye after 5 weeks. Adverse effects, mainly cataract and mild retinal bleeding, were observed, which were probably caused by injection trauma. No histological differences were seen between the treated and untreated eyes. In rabbits, unlabeled hydrogel was injected into the vitreous, and no side effects were observed in the animals. After 3 weeks, the hydrogels could not be seen by fundus imaging, but released dexamethasone was quantifiable with LC-MS/MS in the aqueous humor for 9 weeks post injection. A compartmental model fit of the experimental data showed that the in vivo release of dexamethasone followed first-order kinetics with a half-life of 16.5 days. The good tolerance of the formulation and the sustained dexamethasone release for 2 months make this delivery system an interesting candidate for further preclinical testing.

## Graphical abstract

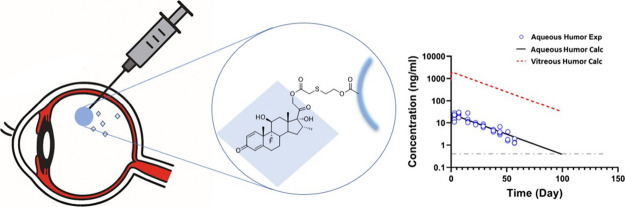

## 1 Introduction

Corticosteroids, such as dexamethasone, are widely used for different age-related, acute, and chronic inflammatory intraocular diseases, including diabetic retinopathy, (diabetic) macular edema, uveitis, retinal vein occlusion, and postoperative inflammation.^1–4^ Due to the short vitreous half-life of ∼3 h of dexamethasone,^5^ dexamethasone is administered intravitreally as a suspension, allowing prolonged drug retention and slow dissolution.^4^ Moreover, an intravitreal implant of poly­(lactic-*co*-glycolic acid) (Ozurdex) is clinically available for sustained dexamethasone release.^6,7^ The implant can be administered in 6-month intervals, but monkey studies showed that dexamethasone release was characterized by a biphasic profile,^8^ namely, a fast release phase for 2 months, resulting in a maximum concentration of dexamethasone of 213 ± 49 ng/mL in the vitreous, followed by a slow release phase. The slow phase results in a dramatic drop in dexamethasone levels 3 months after implantation and low levels of 0.00131 ± 0.0002 ng/mL at 180 days, which is far below the therapeutically effective concentration of dexamethasone (1 nM, or 0.393 ng/mL).^8,9^ Evidently, there is a need for intraocular dexamethasone therapies with better control of drug release. Furthermore, the implantation of Ozurdex with a large 22-gauge needle may cause significant trauma and bleeding in the conjunctiva and sclera as well as retinal damage.^10,11^ Importantly, case studies have shown implant migration into the anterior chamber, which can cause vision-threatening complications if the implant is not surgically removed or relocated.^11–14^ Even though the Ozurdex implant is biodegradable, retention of implant remnants for more than 12 months may lead to vitreal accumulation of polymer materials during multiple dosing regimens.^15^ Ongoing efforts are therefore directed at developing other intravitreal formulations, such as hydrogels and polymeric nanomaterial systems,^16–18^ that are injectable through small (≥30-gauge) needles to minimize the mechanical trauma caused by the injection and provide sustained and consistent drug release for months, associated with full vitreal clearance of the materials.

Previously, we presented an injectable self-healing hydrogel for sustained release of dexamethasone.^16^ The hydrogel consists of an ABA triblock copolymer, with hydrophilic polyethylene glycol (PEG) as the midblock, and outer blocks consisting of thermosensitive *N*-isopropylacrylamide (NIPAM), *N*-acryloxysuccinimide (NAS) used for chemical cross-linking, and a hydrolyzable dexamethasone prodrug with a methacrylate functionality (mDEX). NIPAM-based hydrogels have been previously explored as injectable drug delivery systems for localized drug delivery, as the polymer undergoes physical cross-linking via thermogelation at body temperature.^19–21^ The polymer, p­(NIPAM-*co*-NAS-*co*-mDEX)-PEG-p­(NIPAM-*co*-NAS-*co*-mDEX), abbreviated as PNADEX, was converted into a hydrogel by addition of a disulfide-containing cross-linker, cystamine (CA). The hydrogel was injectable through a small 30-gauge needle post-cross-linking, due to its self-healing properties, which are attributed to the presence of reversible and exchangeable disulfide bonds in the cross-links of the hydrogel.

Moreover, this PNADEX-CA hydrogel showed >400 days release of dexamethasone, and it was cytocompatible with cultured retinal pigment epithelial (ARPE-19) and macrophage (RAW 264.7) cell lines.^16^ In this study, we investigated this hydrogel in vivo in rats and rabbits. We demonstrate prolonged ocular retention and dexamethasone release from the hydrogel, as well as relatively good safety of the materials.

## 2 Materials and Methods

Most chemicals and solvents were obtained from Sigma-Aldrich (Zwijndrecht, The Netherlands) and used as received, unless indicated otherwise. Dichloromethane was obtained from Biosolve (Valkenswaard, The Netherlands), and 4-dimethylaminopyridine (DMAP) was purchased from Fluka (Zwijndrecht, The Netherlands). BDP FL amine, a primary amine derivative of borondipyrromethene, was obtained from Lumiprobe GmbH, Hannover, Germany. Phosphate-buffered saline (PBS) pH 7.4 used for cloud point determination was prepared from concentrated PBS (Fisher BioReagents, Thermo Fischer Scientific, Waltham, MA, USA) and 10 times diluted prior to use with Milli-Q water (final composition: 11.9 mM phosphates, 137 mM sodium chloride, 2.7 mM potassium chloride). Dulbecco’s PBS (pH 7.4), obtained from Gibco, Thermo Fisher, UK, was used for the in vivo experiments. Bovine vitreous was collected from a local slaughterhouse. The Pierce Chromogenic Endotoxin Quant Kit for endotoxin quantification was obtained from Thermo Fischer Scientific (Waltham, MA, USA).

### 2.1 Synthesis of PNADEX Polymer

PNADEX was synthesized as previously reported (Scheme 1).^16^ Briefly, PEG_6000Da_ macro chain transfer agent (CTA) (40 mg, 6.7 μmol, synthesized as previously described^16^), *N*-isopropylacrylamide (NIPAM) (191 mg, 1.69 mmol), *N*-acryloxysuccinimide (NAS) (15 mg, 89 μmol), and a methacrylated dexamethasone prodrug with hydrolyzable thioether functionality, synthesized according to literature^16,19^ (mDEX (26 mg, 44 μmol, structure shown in Scheme 1), were dissolved in 1.5 mL dry *N,N*-dimethylformamide (DMF). Next, 79 μL of azobis­(isobutyronitrile) (AIBN) stock (5 mg/mL in dry DMF) was added (molar ratio 1:0.4 of RAFT macro CTA to AIBN), and the mixture was purged with nitrogen at room temperature. Polymerization was carried out at 70 °C for 24 h under nitrogen atmosphere and constant stirring. The formed polymer was precipitated in ice-cold diethyl ether and collected after centrifugation for 10 min at 4 °C and 10,000*g*. The supernatant was removed, and the remaining solvent was evaporated under reduced pressure. To remove solvent traces of DMF, the crude product was dissolved in and dialyzed against DMSO for 2 days (MWCO 3.5 kDa, Spectra/Por 45 mm, Carl Roth, Karlsruhe, Germany), followed by freeze-drying. The dry polymer was dissolved in endotoxin-free ultrapure water (1 h at 4 °C) and freeze-dried using sterile glassware to obtain a white fluffy powder, with a yield of 77%. Finally, the polymer was characterized by GPC and ^1^H NMR. As a control, a corresponding polymer without mDEX (abbreviated as PNA) was synthesized as described according to a previously published method^16^ and also characterized by GPC and ^1^H NMR.

**Scheme 1:**
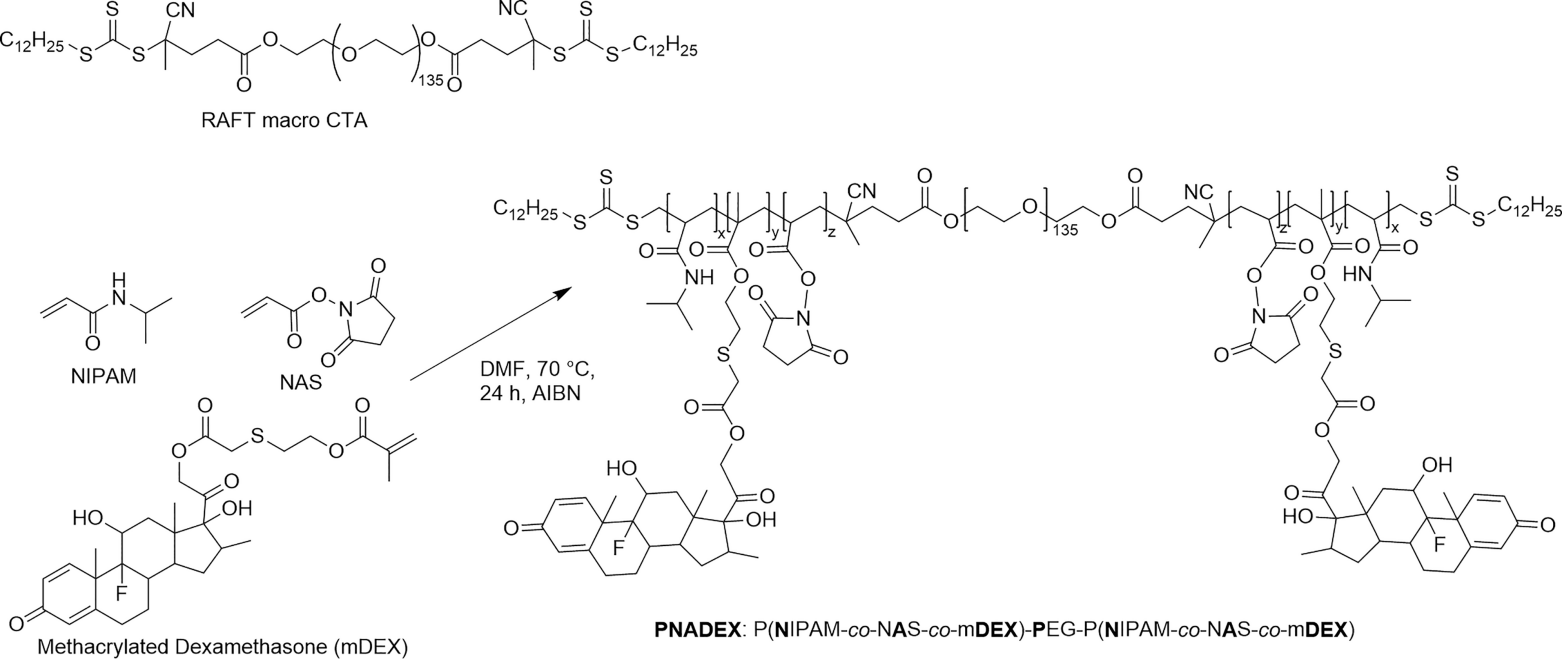
Synthesis Route of PNADEX^16^

### 2.2 Labeling of PNADEX Polymer to Obtain PNADEX-BDP

For labeling the polymer with a primary amine derivative of borondipyrromethene dye (BDP), PNADEX was synthesized as described in Section 2.1 and collected by precipitation in ice-cold diethyl ether, with a yield of 82%. The polymer was analyzed by ^1^H NMR, and 71 mg (2.1 μmol, *M*_n_ 34.4 kDa determined by ^1^H NMR, with 27 μmol equivalent of NHS groups) was dissolved in DMSO (20 mg/mL). Next, 141 μL of a stock solution of 25 mg/mL BDP dye in DMSO (8.2 μmol) was added, and the mixture was left to react overnight at room temperature under constant stirring (NHS to BDP dye molar ratio 3.4:1). Next, the solution was dialyzed against DMSO for 2 days to remove unconjugated dye, and when the dialysate became clear, the polymer was obtained after freeze-drying. The polymer was reconstituted in endotoxin-free water using sterile glassware at 4 °C, dialyzed against endotoxin-free ultrapure water (1 day at 4 °C) to remove traces of DMSO, and freeze-dried. The labeled polymer, abbreviated as PNADEX-BDP, was collected as yellow powder with a yield of 92% and characterized by GPC and ^1^H NMR. The labeling degree (LD) of PNADEX-BDP was determined by evaluating the extinction coefficient at 503 nm (corresponding to the peak UV–vis absorbance of BDP) using a Shimadzu UV-2450 spectrometer and quartz cuvettes with a 10 mm path length (Shimadzu Corporation, Kyoto, Japan). In detail, PNADEX-BDP was first dissolved in DI water at a concentration of 10 mg/mL and subsequently diluted to concentrations of 0, 1.25, 2.5, and 5.0 mg/mL. The absorbance at 503 nm was measured for the polymer solutions, and linear curve fitting and the molecular weight of the polymer were used to determine the extinction coefficient (ε_PNADEX‑BDP_). The determined ε_PNADEX‑BDP_ was compared to the extinction coefficient of BDP dye (ε_BDP_) (92,000 mL mmol^–1^ cm^–1^, reported by Lumiprobe), and the LD was determined according to the equation below, representing the number of dye molecules per polymer chain.

$${\mathrm{LD}}_{\mathrm{BDP}}=\frac{\varepsilon_{\mathrm{PNADEX}\_\mathrm{BDP}}}{\varepsilon_{\mathrm{BDP}}}$$

### 2.3 NMR Spectroscopy

The compositions of the obtained polymers were determined by ^1^H NMR at 400 MHz. NMR spectra were measured with an Agilent 400-MR NMR spectrometer (Agilent Technologies, Santa Clara, USA). Approximately 5 mg of analyte was dissolved in 0.6 mL of DMSO-*d*_6_, and the chemical shifts of analytes were calibrated according to the residual solvent peak visible in the spectra (δ = 2.50 ppm).

### 2.4 Gel Permeation Chromatography (GPC)

Gel permeation chromatography (GPC) was performed to determine the number-average molecular weight (*M*_n_), weight-average molecular weight (*M*_w_), and dispersity (*Đ*= *M*_w_/*M*_n_) of the polymers. A Waters Alliance System (Waters Corporation, Milford, MA, USA) was used with a refractive index and a mixed-D column (Polymer Laboratories) at 65 °C. For PNADEX-BDP, an additional UV detector (detection wavelength: 500 nm) was used. An eluent of 10 mM LiCl in DMF was used as the mobile phase with a flow rate of 1 mL/min. Samples were dissolved in the eluent at a concentration of 3 mg/mL and filtered before analysis over a 0.2 μm PTFE filter (Whatman Mini-UniPrep G2 syringeless filter, Sigma-Aldrich). A series of linear PEGs with narrow and defined molecular weights (PSS GmbH, Mainz, Germany) were used as calibration standards.

### 2.5 Determination of Cloud Point

The cloud point (CP) of PNADEX was measured with a Jasco FP8300 spectrofluorometer (Tokyo, JP). The polymer was dissolved in PBS at a concentration of 3 mg/mL at 4 °C. The temperature was increased from 4 to 45 °C at a rate of 1 °C/min. The scattering intensity was measured at 650 nm, and the CP was defined as the onset of increasing scattering intensity.^23^

### 2.6 Endotoxin Quantification

Endotoxin levels of the polymers were quantified using the Pierce Chromogenic Endotoxin Quant Kit (Thermo Fischer Scientific, Waltham, MA, USA), according to the manufacturer’s instructions. The polymers were dissolved in endotoxin-free water to a concentration 5 mg/mL and further diluted to 2.5 and 0.5 mg/mL (*n* = 2). Endotoxin levels of 100 μL samples were measured in a 96-well plate against endotoxin standards prepared in duplicate according to the kit specifications, with a linear range of 0.1–1.0 EU/mL, at 37 °C. The results were analyzed using Microsoft Excel 2016 and GraphPad Prism version 9.1.

### 2.7 Formation of 10 wt % Hydrogels for Injection in Rats and Rabbits

The hydrogels for the animal experiments were formed inside a BD Micro-Fine +0.3 mL insulin syringe with a 30G needle (pip code 230–4533, BD, Franklin lakes, NJ, USA). For the experiments in rats, 18.5 mg of fluorescently labeled PNADEX-BDP was dissolved in 148 μL of sterile PBS overnight at 4 °C. Next, 18.5 μL of cystamine dihydrochloride salt (CA) stock solution (48 mg/mL) in sterile filtered borate buffered saline (BBS) (9.0 g/L NaCl, 6.0 g/L Na_2_B_4_O_7_, 7.4 g/L H_3_BO_3_, pH 9.0) was prepared, added to the polymer solution (molar ratio of NHS: amine group in CA in the final mixture was 1:1), mixed thoroughly, and the resulting solution was aliquoted aseptically in 20 μL volumes into the syringes with the plunger removed. The plunger was carefully inserted, and the syringe was placed into 50 mL falcon tubes to prevent evaporation. The syringes were subsequently incubated at 37 °C for 3 h to allow gel formation. Prior to injection, excess material was extruded from the syringe to perform 5 μL injections into the eyes of the animals.

For the experiments with rabbits, unlabeled PNADEX-CA and PNA-CA gels were prepared inside the insulin syringes as described above. Briefly, 41.8 mg of unlabeled PNADEX was dissolved in 334 μL of PBS and mixed with 42 μL of 59 mg/mL CA stock. For blank hydrogels without dexamethasone, 46.3 mg of unlabeled PNA was dissolved in 370 μL of PBS and mixed with 46 μL of 45 mg/mL CA stock. The resulting 10 wt % polymeric solutions with cross-linker were aliquoted à 70 μL into insulin syringes, followed by incubation at 37 °C for 3 h. Prior to injections, excess material was extruded out, and 50 μL intravitreal injections were performed into the eyes of the animals. For the unlabeled PNADEX-CA hydrogels, the injected dose corresponded to 402 μg of dexamethasone.

### 2.8 In Vivo Studies

The safety of the intravitreally injected fluorescently labeled PNADEX-BDP-CA hydrogel was evaluated first in rats, followed by pharmacokinetic evaluation of unlabeled PNADEX-CA in rabbits, and safety evaluation of unlabeled PNADEX-CA and PNA-CA hydrogels in rabbits. All animal experiments were carried out in accordance with the ARVO guidelines for the use of animals in ophthalmic and vision research and followed the 3R principle of replacement, reduction, and refinement.^24^ The procedures were approved by the Finnish National Project Authorization Board and were supervised by the Animal Welfare Board of the Laboratory Animal Center of the University of Eastern Finland (License number ESAVI-2020-027769).

#### 2.8.1 Safety Evaluation in Rats

Four 3-month old male Lister hooded rats (HsdOla:LH) were used for the safety evaluation of the formulation. Three animals received 10 wt % fluorescently labeled PNADEX-BDP-CA hydrogel formulation in both eyes, while one animal received injection only in one eye, with the contralateral eye of this animal serving as a control (*n* = 7 injected eyes). The animals were kept under a 12-h light/dark cycle and housed postoperation alone in individually ventilated cages, with food and water provided ad libitum. The cages were provided with sufficient bedding, nesting material, and enrichment.

##### 2.8.1.1 Intravitreal Injections

Twenty μL of 10 wt % PNADEX_BDP-CA hydrogel was formed as described in Section 2.7. Intravitreal injections of 5 μL were performed into the rats using an insulin syringe with a 30 G needle. Injections were administered approximately 1 mm from the limbus at a 45° angle toward the back of the vitreous. Anesthesia was induced with 4% isoflurane (Attane Vet 1000 mg/mL, Piramal Critical Care B.V., Voorschoten, The Netherlands) with a 500 mL/min air flow and maintained with 2% isoflurane and a 250 mL/min air flow. The pupils of the rats were dilated 15 min prior to injections and eye imaging by applying 10 μL of topical tropicamide to each eye (Oftan Tropicamid 5 mg/mL, Santen Pharmaceutical Co., Tampere, Finland). Local ocular surface anesthesia before intravitreal injections was achieved by applying 10 μL of topical oxybuprocaine (Oftan Obucain, 4 mg/mL; Santen Pharmaceutical Co., Tampere, Finland). A topical ocular carbomer hydrogel was applied after intravitreal injections and during imaging to prevent corneal dryness (Viscotears, 2 mg/g, Dr. Winzer Pharma, Berlin, Germany). Chloramphenicol eye cream (Oftan Chlora 10 mg/g, Santen Pharmaceutical Co., Tampere, Finland) was applied topically after intravitreal injections.

##### 2.8.1.2 Fundus and OCT Imaging

Eye imaging was carried out at baseline, immediately after injection, 1 day and 4 days after injection, and once weekly until 5 weeks post-injection using OCT and a fundus camera (Phoenix MICRON MICRON IV/OCT, CA, USA). For eye imaging up to 3 weeks, the animals were sedated with isoflurane anesthesia as described above. Prior to imaging, the ocular muscles were relaxed with topical medetomidine (10 μL, Domitor vet 1 mg/mL). Due to the animals developing tolerance over time against isoflurane anesthesia, the animals were anesthetized prior imaging at 4 and 5 weeks after hydrogel administration with subcutaneous medetomidine (0.4 mg/kg, Domitor vet 1 mg/mL, Orion Pharma, Espoo, Finland) and ketamine (60 mg/kg, Ketalar/Ketaminol vet 50 mg/mL, Pfizer Oy Animal Health, Espoo, Finland), and anesthesia was reversed by subcutaneous atipamezole (1.25 mg/kg, Antisedan vet 5 mg/mL, Orion, Finland). OCT imaging was used to noninvasively evaluate retinal integrity for signs of retinal detachment, subretinal fluid, holes, or any other abnormalities in the retina as well as signs of neovascularization. Fundus images were used to follow the degradation of the hydrogel and to evaluate any signs of retinal neovascularization, hemorrhages, or any other abnormalities.

##### 2.8.1.3 Histological Evaluation

The animals were sacrificed 6 weeks post-injection by overdose of carbon dioxide, followed by cervical dislocation. The eyes were collected and post-fixated overnight in 4% paraformaldehyde (PFA). The following day, the eyes were processed for paraffin embedding in a processing machine (Shandon Citadel 2000 Tissue Processor, Thermo Fischer Scientific, USA) as described in the Supporting Information (SI Table S1). The eyes were cast in liquid paraffin, and 6 μm vertical cross-sections were prepared from the proximity of the optic nerve for histological analysis. The sections were stained using a standard hematoxylin eosin (H&E) staining procedure (SI Table S2) and imaged using a Zeiss light microscope (Axio Imager M2 with AxioCam MRm, Zeiss, Germany) with 20× magnification (EC Plan-NEOFLUAR 20*X*/0.5 objective, Zeiss, Germany). The outer nuclear layer (ONL) thickness was measured using ImageJ software.^25,26^ For analysis, five images of each eye were analyzed, and five measurements were taken for each image.

#### 2.8.2 Pharmacokinetics and Safety Evaluation in Rabbits

For evaluation of safety and dexamethasone delivery with the 10 wt % unlabeled PNADEX-CA hydrogel, three 3-month-old female New Zealand rabbits (weight 2.6–3.0 kg) were used. As a control in the safety assessment, three rabbits (weight 2.0–2.4 kg) received the 10 wt % unlabeled PNA-CA hydrogel into their eyes. The animals were individually housed in standard laboratory rabbit cages and were fed a standard diet. Food and water were provided ad libitum. At the end of the study (week 9 for the animals injected with the PNADEX-CA hydrogel and week 4 for animals treated with the PNA-CA hydrogel), the animals were sacrificed by injecting a lethal dose of pentobarbitone (Mebunat vet 60 mg/mL, Orion, Finland) into the marginal ear vein.

##### 2.8.2.1 Intravitreal Injections

Seventy μL of 10% PNADEX-CA or PNA-CA hydrogels were formed as described in Section 2.7, and 50 μL intravitreal injections were performed into the rabbit eyes using an insulin syringe with a 30 G needle. In the group receiving PNADEX-CA hydrogel, two animals received the hydrogel formulation in both eyes, while one animal received the injection only in one eye (*n* = 5). In the group receiving PNA-CA hydrogel, three animals received the hydrogel formulation in both eyes (*n* = 6). The injection was done about 4 mm from the limbus trans-sclerally into the vitreous within 5 s. Anesthesia was achieved by subcutaneous medetomidine (0.5 mg/kg, Domitor vet 1 mg/mL, Orion Pharma, Espoo, Finland) and ketamine (25 mg/kg, Ketaminol, 50 mg/mL; Pfizer Animal Health, Espoo, Finland). The pupils were dilated 15 min prior to injection by topical tropicamide, and ocular surface anesthesia was achieved with oxybuprocaine eye drops a few minutes before the intravitreal injections. Immediately after the injections, a carbomer hydrogel (Viscotears 2 mg/g, Alcon/Dr. Gerhard Mann chem.-pharm. Fabrik GmbH, Berlin, Germany) was applied onto the eyes to prevent corneal dryness. Anesthesia was reversed by sc injection of atipamezole (0.2 mL/kg; Antisedan, 5 mg/mL; Orion Pharma, Espoo, Finland).

##### 2.8.2.2 Aqueous Humor Collection

Animals that received PNADEX-CA hydrogel into their eyes were anesthetized as described in Section 2.8.2. Aqueous humor samples were withdrawn using a 34G needle through the limbus for dexamethasone quantification. This method was chosen over direct sampling from the vitreous as the latter is a very invasive approach. After sampling, the eyes were treated with antibiotic ointment (Oftan Chlora 10 mg/g), and the animals received an additional painkiller s.c. (carprofen, 4 mg/kg, Rimadyl vet 50 mg/mL, Zoetis Animal Health ApS, Copenhagen, Denmark). Anesthesia was reversed by atipamezole (s.c). The samples were stored at −80 °C until analysis.

##### 2.8.2.3 Safety Evaluation

Fundus imaging was carried out before hydrogel injection, immediately after, 3 days, and 1-week post-injection of unlabeled PNADEX-CA or PNA-CA hydrogels, further followed by weekly imaging. For rabbits that received the PNA-CA hydrogel, imaging was carried out for 3 weeks, whereas for the animals that received the PNADEX-CA hydrogel, imaging was caried out for 2 months. In addition, slit lamp evaluation was performed by an ophthalmologist during the experiment to evaluate any adverse effects caused by the hydrogel formulations. The slit lamp evaluation was done prior to injection and 9 days post-injection for animals treated with the PNA-CA hydrogel, and 21 and 60 days post-injection for animals that received the PNADEX-CA hydrogel.

##### 2.8.2.4 Mass Spectrometry

Aqueous humor samples (50–80 μL) were thawed and adjusted to 150 μL with blank bovine aqueous humor. The samples were spiked with 10 μL of internal standard (ISTD) solution of 100 ng/mL deuterium-labeled dexamethasone (dexamethasone-*d*_5_, Toronto Research Chemicals, Ontario, Canada) in 30% acetonitrile in water). One mL of methyl *t*-butyl ether was added to each sample, followed by shaking at room temperature for 10 min. Phases were allowed to separate for 30 min, and the organic phase containing the extracted dexamethasone was transferred into glass vials, and the solvent was evaporated in a vacuum centrifuge (Savant SpeedVac Concentrator, Thermo Fischer Scientific). The dried residue was solubilized in 50 μL of 30% acetonitrile in water.

Dexamethasone calibration standards (0.01–500 ng/mL) were prepared in duplicate, and quality controls (three levels in the range of 0.25 −250 ng/mL) were prepared in triplicate using bovine aqueous humor as the matrix and processed thereafter similarly as samples.

Dexamethasone concentrations were measured with LC–MS/MS (Agilent 1290 series liquid chromatograph and an Agilent 6495 triple-quadruple mass spectrometer, Agilent Technologies, Inc., USA). The analytes were separated with a reversed-phase column (Poroshell 120 SB-C18, 2.1 mm × 50 mm, 2.7 μm, Agilent) at 50 °C. The aqueous mobile phase (A) was 0.1% formic acid in Milli-Q water, and the organic mobile phase (B) was methanol. The following gradient was used: 0.0 to 2.5 min: 30% → 100% B, 2.5 to 3.0 min: 100% B, 3.0 to 3.1 min: 100% → 30% B, 3.1 to 4.5 min 30% B. The solvent flow rate was 0.5 mL/min, and the injection volume was 2 or 10 μL. The following ion source conditions were employed: sheath gas heat 350 °C, drying gas temperature 200 °C, drying gas flow 16 L/min, nebulizer pressure 25 psi, and capillary voltage 4000 V. Detection was based on multiple reaction monitoring (MRM), and positive electrospray ionization mode was used. Transitions monitored were *m*/*z* 393.0 → *m*/*z* 373.1 (CE 4) and *m*/*z* 393.0 → *m*/*z* 355.0 (CE 12) for dexamethasone, and *m*/*z* 398.0 → *m*/*z* 378.1 (CE 8) and *m*/*z* 398.0 → *m*/*z* 360.2 (CE 8) for dexamethasone-*d*_5_. The data were analyzed with Agilent Mass Hunter Quantitative Analyzed software (vB.09.00, build 9.0.647.0, Agilent Technologies, CA, USA). The lower limit of quantitation (LLOQ) was 0.5 ng/mL. The accuracy, expressed as the deviation from the nominal concentration, and the precision (relative standard deviation, RSD) were ≤ 20% for the calibration and QC sample levels.

### 2.9 Pharmacokinetic Simulation

A compartmental model was used to calculate the release rate of dexamethasone from the hydrogel (Figure 1). The model assumes that dexamethasone in the hydrogel is quantitatively releasable, and that neither metabolism nor chemical degradation of the drug takes place. The model further assumes that neither hydrogel matrix degradation nor ocular clearance of the polymeric materials occurs. Moreover, both transretinal and anterior elimination routes of dexamethasone were included.^27,28^ The compartmental model included the following differential equations:

$$\frac{dM_{\mathrm{gel}}}{dt}=-k_{r}M_{\mathrm{gel}}$$ 1

$$\frac{dM_{\mathrm{vit}}}{dt}=k_{r}M_{\mathrm{gel}}-k_{\mathrm{vit}}M_{\mathrm{vit}}$$ 2.1

Note that *k*_vit_ = *k*_p_ + *k*_ant_; thus,

$$\frac{dM_{\mathrm{vit}}}{dt}=k_{r}M_{\mathrm{gel}}-(k_{p}+k_{\mathrm{ant}})M_{\mathrm{vit}}$$ 2.2

or:

$$\frac{dM_{\mathrm{vit}}}{dt}=k_{r}M_{\mathrm{gel}}-k_{p}M_{\mathrm{vit}}-k_{\mathrm{ant}}M_{\mathrm{vit}}$$ 2.3

**Figure 1:**
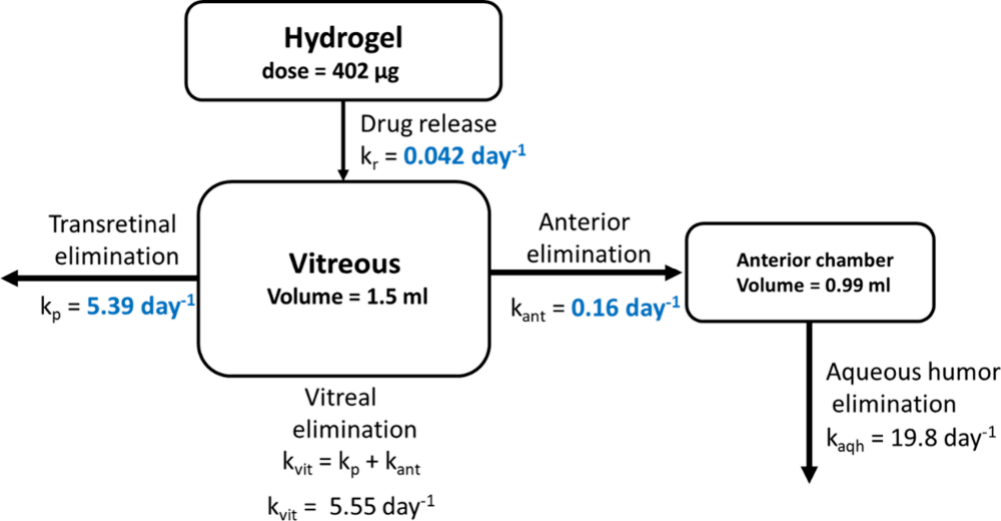
Compartmental pharmacokinetic model of dexamethasone release from PNADEX-CA hydrogel after injection into rabbit vitreous. Note that the values in blue are considered the result of the curve fitting, where *k*_ant_ and *k*_r_ were used as floating parameters, and *k*_p_ was inferred based on *k*_vit_ and *k*_ant_.

Note that *k*_p_ = *k*_vit_ – *k*_ant_. Hence, we can eliminate the constant *k*_p_:

$$\frac{dM_{\mathrm{vit}}}{dt}=k_{r}M_{\mathrm{gel}}-(k_{\mathrm{vit}}-k_{\mathrm{ant}})M_{\mathrm{vit}}-k_{\mathrm{ant}}M_{\mathrm{vit}}$$ 2.4

$$\frac{dM_{\mathrm{aqh}}}{dt}=k_{\mathrm{ant}}M_{\mathrm{vit}}-k_{\mathrm{aqh}}M_{\mathrm{aqh}}$$ 3

The model was built using published experimental values for dexamethasone: vitreal elimination rate constant (*k*_vit_ = 5.55 day ^–1^), intravitreal volume of distribution (*V*_dvit_ = 1.5 mL), intracameral clearance (13.6 μL/min), anterior chamber volume of distribution (*V*_dant_ = 0.99 mL), and elimination rate constant from the anterior chamber (*k*_aqh_ = 19.8 day^–1^).^5,29^ *M*_gel_, *M*_vit_, and *M*_aqh_ are the mass of dexamethasone in the hydrogel, vitreous, and aqueous humor, respectively. *M*_gel_ is the dose of loaded dexamethasone in the injected hydrogel (402 μg). *M*_vit_ and *M*_aqh_ were obtained by multiplying the measured concentration of dexamethasone by the vitreous (*V*_dvit_ = 1.5 mL) and aqueous humor volume of distribution (*V*_dant_ = 0.99 mL), respectively. The parameters *k*_p,_ *k*_ant_, and *k*_r_ are the rate constants for transretinal vitreous elimination, elimination of dexamethasone from the vitreous to the anterior chamber, and dexamethasone release rate constant from the hydrogel, respectively. The experimental concentrations in the aqueous humor were fitted in the equations, considering *k*_ant_ and *k*_r_ as floating parameters. R programming language was used for curve fitting.^30–32^ The experimental data were fitted to the model using the least-square method by the Levenberg–Marquardt algorithm.^33^ Pharmacokinetic simulations using the derived parameters were carried out using STELLA software (v. 8.1.1) (ISEE systems, USA) with the fourth-order Runge–Kutta algorithm.

## 3 Results and Discussion

### 3.1 Characterization of Synthesized Polymers

RAFT polymerization was used to synthesize an ABA triblock copolymer abbreviated as PNADEX, containing PEG as the midblock with a thermosensitive outer block consisting of NIPAM, NAS, and mDEX. Figure 2 shows the structures of PNADEX and its BDP-labeled variant (PNADEX-BDP). The BDP dye was chemically conjugated to the NHS-activated acrylic acid units of the polymer, which were also used for chemical cross-linking with cystamine. For simplicity, one dye molecule is shown in PNADEX-BDP (labeling degree was 7.9%, corresponding to 1 dye molecule per 13 polymer chains; Supporting Information). Low coupling efficiency of approximately 2% of the BDP dye (Supporting Information) can be explained by the reaction conditions. The coupling was carried out in DMSO, and without addition of a base, the dye in HCl salt form has low reactivity. Additionally, a triblock copolymer lacking mDEX (abbreviated as PNA) was synthesized. The copolymer compositions as determined by ^1^H NMR and other characteristics of the polymers are presented in Table 1. Importantly, the copolymer compositions of the thermosensitive blocks (NIPAM:NAS:mDEX molar ratios) as determined by NMR were close to that of the feed. The ^1^H NMR spectra of polymers are presented in the Supporting Information (Figures S1–S3). The *M*_n_ determined from ^1^H NMR were systematically higher (32.1, 35.3, and 30.1 kDa for PNADEX, PNADEX-BDP, and PNA, respectively) than *M*_n_ values measured with GPC (14.5, 12.4, and 18.5 kDa for PNADEX, PNADEX-BDP, and PNA, respectively). The GPC chromatograms are presented in Supporting Information, Figure S3. The lower *M*_n_ values determined by GPC may be due the use of PEGs, which have high hydrodynamic volume as calibration standards^34–36^ and is in line with previous reports for these polymers.^16^ The molecular weight distribution, as reflected by their *M*_w_/*M*_n_ values of 1.4–1.7, and cloud points of 23–32 °C were also in line with previously reported values.^16^ GPC analysis did not detect the presence of unconjugated BDP dye in PNADEX-BDP samples (Supporting Information, Figure S5).

**Figure 2:**
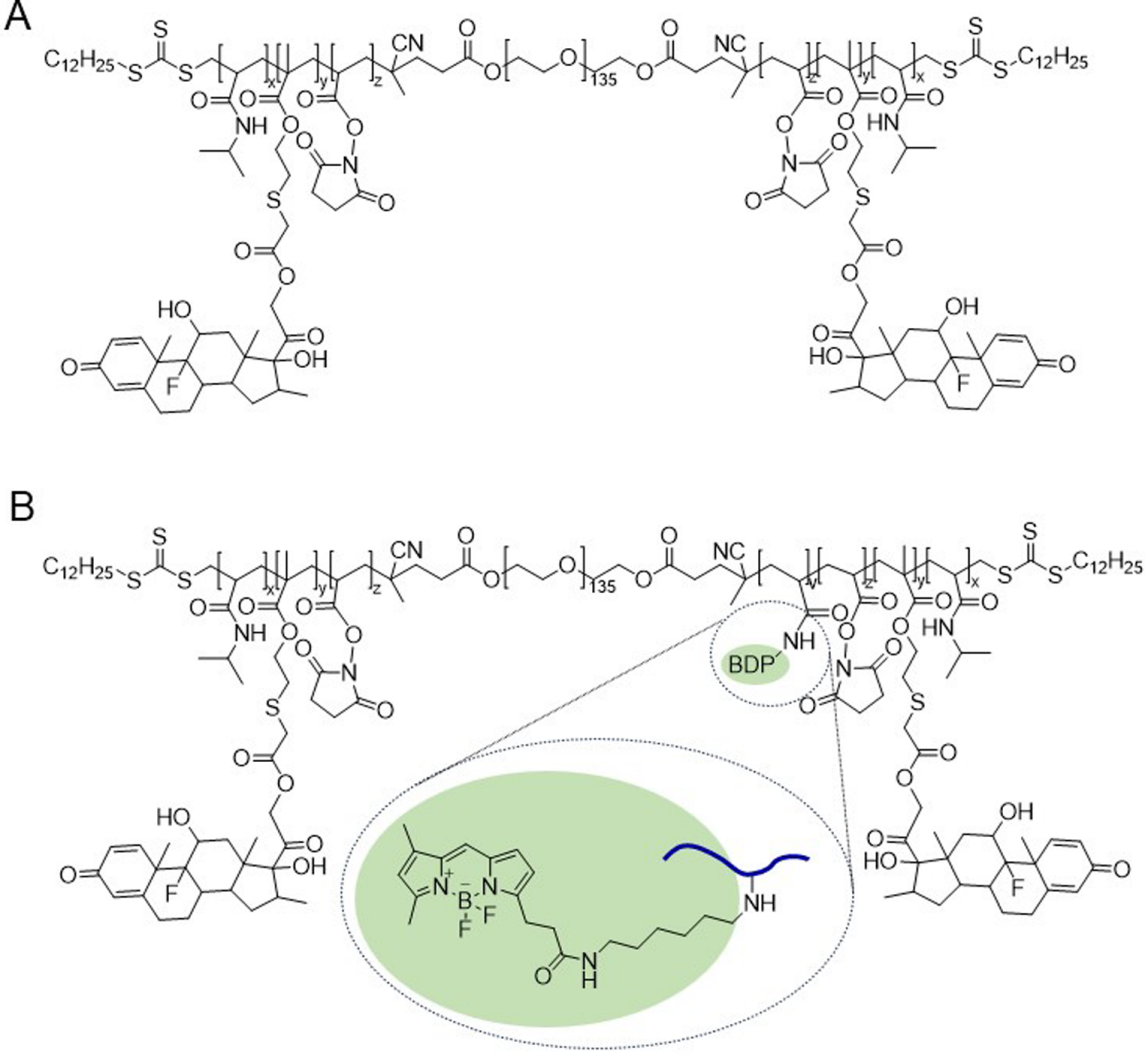
Chemical structures of PNADEX (A) and PNADEX-BDP (B). For simplicity, only one covalently linked BDP dye is shown.

**Table 1:** Characteristics of PNADEX, PNADEX-BDP, and PNA Polymers

polymer	feed ratio NIPAM:NAS:mDEX	copolymer composition^a^ NIPAM:NAS:mDEX	*M*_n_ ^a^ (kDa)	*M*_n_ ^b^ (kDa)	*Đ* ^b^	CP (°C)	yield (%)
PNADEX	82:7:11	75:11:14	32.1	14.5	1.5	23	77
PNADEX-BDP	82:7:11	80:9:11	35.3	12.4	1.7	23	92
PNA	92:8:0	91:9:0	30.1	18.5	1.4	32	69

a Determined by ^1^H NMR; spectra are shown in the Supporting Information, Figures S1–S3.

b *M*_w_/*M*_n_ as determined by GPC using PEG calibration standards; GPC chromatograms are shown in the Supporting Information, Figure S4.

Each rabbit eye received a 50 μL injection of a 10 wt % preformed hydrogel into the vitreous (volume 1.5 mL).^37^ After the injection, polymer concentration was estimated as 3.33 mg/mL (i.e 5 mg/1.5 mL). Endotoxin levels of PNADEX, PNADEX-BDP, and PNA samples (results shown in Figure 3) were within the Food and Drug Administration (FDA) recommendations for single-use intraocular ophthalmic devices (≤0.2 EU/mL).^38^ The polymer concentrations tested (0.5–5 mg/mL) covered the expected concentration in the vitreous (3.33 mg/mL).

**Figure 3:**
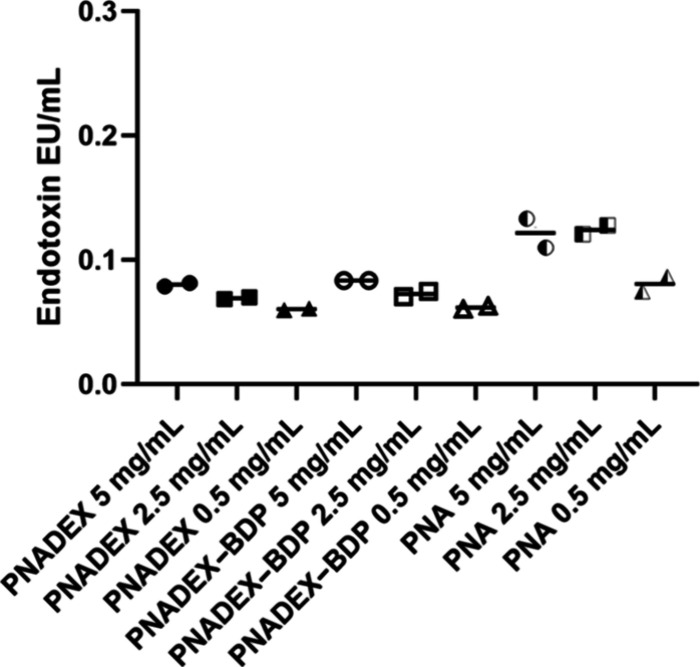
Endotoxin levels of formulations of PNADEX, PNADEX-BDP, and PNA (concentrations ranging from 0.5 to 5 mg/mL). *n* = 2 for all conditions.

### 3.2 Safety Evaluation in Rats

OCT and fundus images were used to visualize the injected hydrogel formulation in the vitreous and to evaluate the safety of the formulation and injection procedure Labeled hydrogel was used in the experiment to confirm the colocalization of the fluorescent signal with the hydrogel detected by OCT. While OCT has been previously used for characterization of hydrogel geometry in vitro and in bioprinting applications, as well as in studying the ocular distribution of liposomes and polymeric nanoparticles in vivo,^39–41^ the method has not been used to follow the shape of intravitreally administered hydrogels in time.

Figure 4 shows fundus and OCT images of three rat eyes (A–C) before injection at the baseline and after a 5 μL injection of 10 wt % PNADEX-BDP-CA hydrogel at different time points (immediately after injection, 1 day post-injection, 4 days post-injection, and 2-, 3-, 4-, and 5-weeks post-injection). During the 5 weeks, the polymer matrix gradually degraded, as indicated by the diminishing green dye. At the baseline, the lens can be seen as a faint concave shape in the upper part of the OCT images, indicated by white arrows. In the fundus images, the hydrogels could be visualized as green turbid material after injection into the vitreous, and the gradual degradation of the matrix can be seen as the intensity of the green signal diminishes. Some details such as air bubbles (indicated by white circles) and refined gel edges could be observed for 2 weeks post-injection in the fundus and OCT images. Thereafter, the hydrogels started to lose their structural integrity, and at week 3, only remnants were seen as faint white shadows in OCT images in the vitreous space below the lens (indicated by green arrows in Figure 4). By week 4, the vitreous space of animals B and C was completely devoid of hydrogel, whereas some gel remnants were still seen in OCT images of animal A. At 5 weeks, the green fluorescent signal was only detected in the optic nerve area (Supporting Information, Figure S7). This accumulation of small polymeric fragments in the optic nerve is in line with previous observations with intravitreal liposomes.^27^ More investigations are needed to evaluate potential long-term effects of hydrogel fragments/degradation products in the optic nerve.

**Figure 4:**
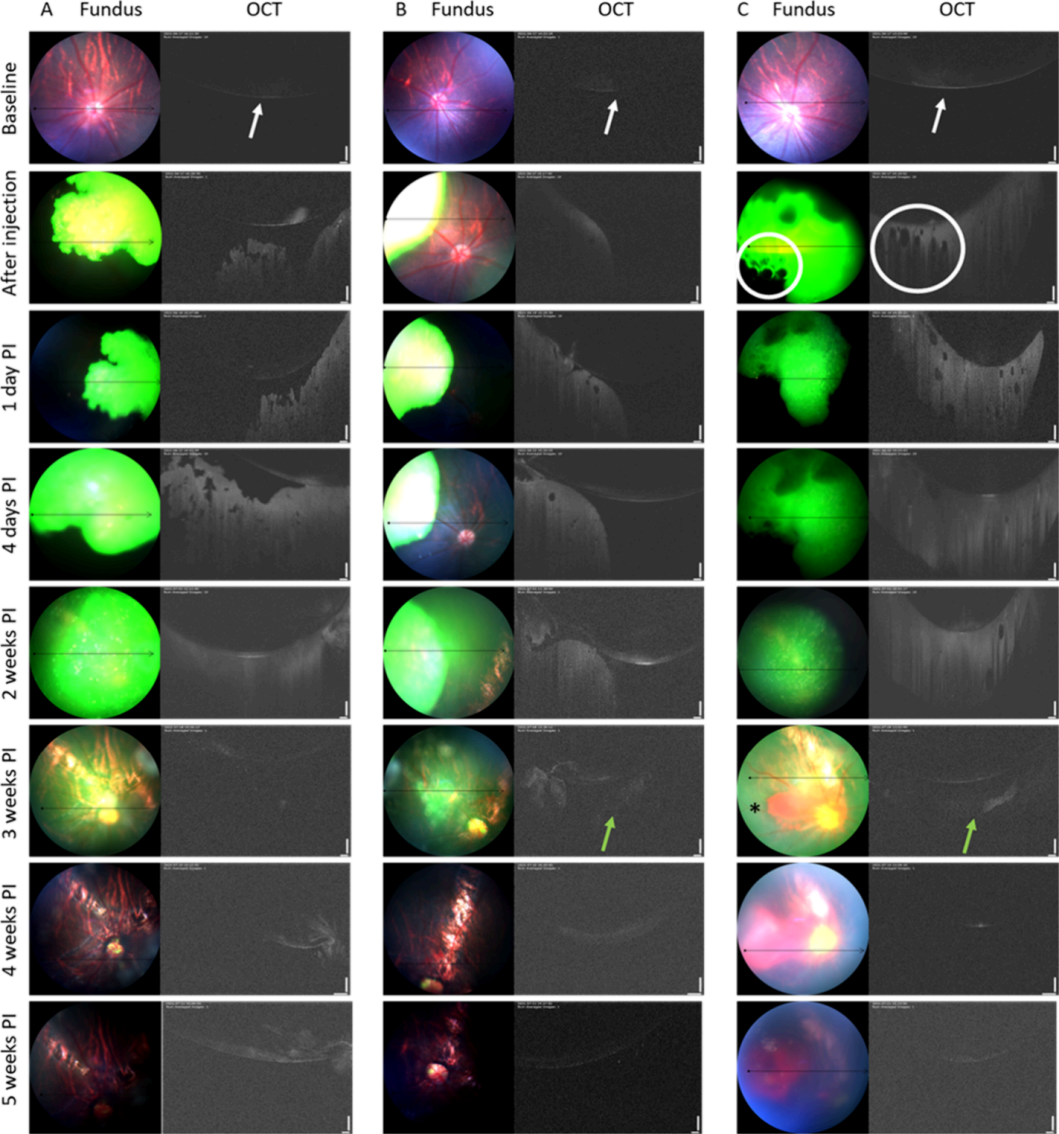
Fundus and OCT images show PNADEX-BDP hydrogel degradation in the vitreous of three individual rat eyes (A–C). Fluorescently labeled hydrogel is seen as green mass in the fundus images, whereas the hydrogel appears as white mass in the OCT images. In the OCT images taken at baseline, the lens can be seen as a white concave shape, indicated by white arrows. Minor trauma can be seen in the fundus images of animal C at 3 weeks post-injection, indicated by star (*).

In 4 out of the 7 injected rat eyes, cataract formation was observed 2–4 weeks after injection. This was probably due to trauma caused to the lens during injections into small rat eyes. It has indeed been reported in the literature that mechanical trauma to the lens, such as the needle touching the lens during intravitreal injection, leads to cataract formation, also known as traumatic cataract.^42–46^ Some retinal damage, likely due to injection trauma, was observed at 3 weeks postinjection in rat C (indicated by * in Figure 4). Cataract prevented adequate fundus and OCT imaging at later times; therefore, only the rat eyes without cataract were imaged for 5 weeks. Importantly, cataract was not observed after intravitreal injection of the hydrogels into the rabbit vitreous, most likely because injections into the larger rabbit eyes are a safer procedure.

Our aim was to use OCT imaging for evaluation of both retinal compatibility and degradation of the hydrogel in the vitreous, but turbidity of the hydrogel and cataract obscured the quality of the retinal OCT images. Therefore, histology was used for further evaluation of the formulation safety. No changes in retinal morphology in treated eyes (Figure 5A) as compared to untreated controls (Figure 5B) were observed. Moreover, we did not observe a statistically significant difference in the outer nuclear layer (ONL) thickness of treated and untreated eyes (37.7 ± 5.2 and 42.1 ± 1.2 μm, respectively, Figure 5C). However, due to the limited sample size, further evaluation of the safety of the formulation is needed, especially in regard of repeated administration. One histological sample (not shown) was excluded from the analysis due to damage incurred during tissue processing.

**Figure 5:**
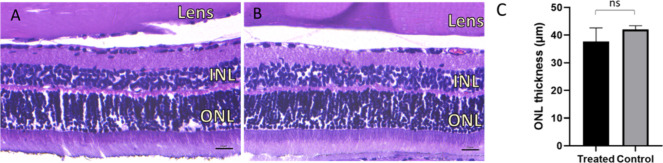
(A) Retinal morphology of the rat eye injected with 5 μL of 10 wt % PNADEX-BDP-CA hydrogel and (B) morphology of the untreated control eye. (C) Outer nuclear layer thickness in treated and untreated control eyes. For analysis, five images of each eye were analyzed, see Section 2.8.1. Unpaired *t* test, no significant difference, *p* > 0.05. *n* = 30 analyzed images for treated and *n* = 5 for untreated. INL= Inner nuclear layer, ONL= outer nuclear layer. Scale bar: 20 μm.

### 3.3 Pharmacokinetics and Safety in Rabbits

For kinetic analysis, 50 μL of PNADEX-CA hydrogel (corresponding to a dose of 402 μg of dexamethasone) was injected into the rabbit vitreous (*n* = 5) and followed as long as dexamethasone levels could be detected. Figure 6 shows an example of fundus images of a representative rabbit eye injected with the PNADEX-CA hydrogel at different times after the injection. The top row shows that adverse effects were observed in neither the retina nor optic nerve areas, and the bottom row shows the gradual degradation of the hydrogel. The slit lamp evaluation carried out 21 and 60 days post-injection did not reveal any conjunctival redness, chemosis, corneal endothelium deposits, cells in the intracameral or vitreous body, nor corneal surface dryness or lid margin redness. Since dexamethasone as an anti-inflammatory drug could mask signs of irritation, 50 μL of blank PNA-CA hydrogel was also injected into the eyes of three rabbits (*n* = 6). Retinal status and hydrogel degradation were followed for 3 weeks. No adverse effects were observed with the PNA-CA hydrogel, indicating that dexamethasone released from the drug-containing PNADEX-CA hydrogel did not mask possible inflammation caused by the hydrogel.

**Figure 6:**
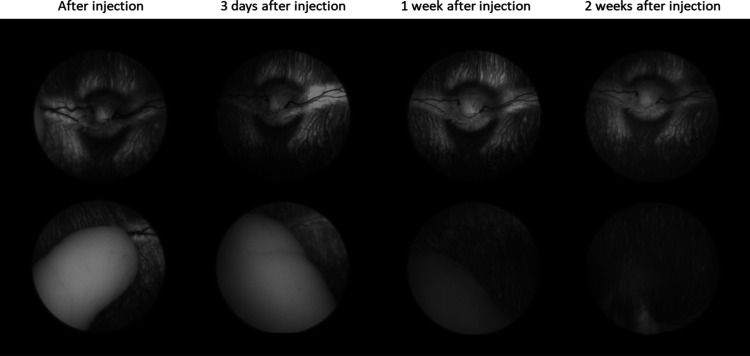
Fundus imaging of 50 μL of PNADEX-CA hydrogel after injection into the rabbit vitreous.

In the case of rats, a 5 μL intravitreal injection fills ≈38% of the 13 μL vitreous volume.^47^ On the contrary, the hydrogel was localized in the periphery of the rabbit vitreous, remaining outside of the visual path. In rabbits, a 50 μL injection fills around 3% of the vitreous cavity of 1.5 mL; hence, the expected hindrance to vision caused by the turbid material is expected to be minimal. Nevertheless, minimizing the size of the delivery system is desirable.

The dexamethasone concentrations in the different samples were quantified by LC–MS/MS as described in Section 2.8.2, and the aqueous humor concentrations of dexamethasone are plotted against time in Figure 7. The peak concentration (*C*_max_) of 22.8 ± 5.0 ng/mL was observed 7 days after the injection, and the levels gradually decreased to 1.4 ± 0.3 ng/mL at day 57 (Supporting Information, Table S3). Hereafter, the concentrations of dexamethasone in the aqueous humor samples were below the lower limit of quantitation (LLOQ) of 0.5 ng/mL.

**Figure 7:**
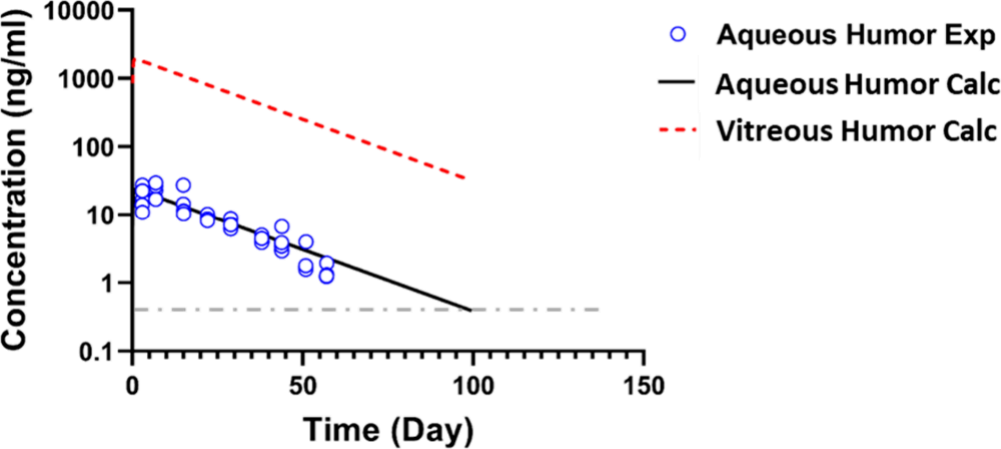
Dexamethasone release from 50 μL of 10 wt % PNADEX-CA hydrogel in the rabbit eye. The measured dexamethasone concentration in the aqueous humor (Aqueous Humor Exp) was used to calculate the values over time in the aqueous humor (Aqueous Humor Calc) and vitreous humor (Vitreous Calc). The calculated curves are derived from the compartmental model shown in Figure 1. The experimental data are presented as individual data points. The therapeutic threshold for dexamethasone in the vitreous (0.393 ng/mL^8,9^ ) is presented as gray dashed line.

Pharmacokinetic modeling (Section 2.9) was used to describe dexamethasone release in vivo and overall pharmacokinetics of PNADEX-CA hydrogels. The curve fitting of dexamethasone concentrations over time is shown in Figure 7. Based on the simulations (Section 2.9), dexamethasone was released from the hydrogel at a first-order rate with a release rate constant (*k*_r_) of 0.042 day^–1^, corresponding to a release half-life of 16.5 days. Dexamethasone was distributed from the vitreous to the aqueous humor at a rate of 0.16 day^–1^. These values were further used in the kinetic simulations to calculate the dexamethasone concentration in the vitreous humor in time.

Unlabeled PNADEX-CA hydrogel was not seen in fundus images 2 weeks after injection, but dexamethasone was quantified with LC/MS-MS for more than 50 days in the aqueous humor (results shown in Figure 7). Previously, it was shown that the elimination half-life of fluorescently labeled dextrans of 10 and 160 kDa were 3.5 and 6.9 days, respectively.^48^ This may explain why dexamethasone release from the PNADEX-CA hydrogel, consisting of polymers of 32–35 kDa, could be quantified for many weeks even after the hydrogels were degrading. Probably, polymer chains formed after degradation of the hydrogel are slowly eliminated from the vitreous. These soluble polymer chains are not visible in OCT, but they are still susceptible to hydrolytic release of dexamethasone. The half-life of intravitreally administered free dexamethasone in the rabbit vitreous is approximately 3 h,^5^ but drug release from the hydrogel and polymer chains retains dexamethasone concentrations in the vitreous for a substantially longer time (Figure 7).

The dexamethasone concentrations in the anterior chamber and vitreous were modeled for 100 days (Figure 7), which is approximately the duration of 6 release half-lives (6 * 16.5 days = 99 days). At this time point, less than 2% of the original dose is expected to remain in the delivery system. The area under the concentration time curve (AUC) from day 0 to day 57 (AUC_0–57d_) in the vitreous compartment for the released dexamethasone corresponded to 91% of the initial dose of dexamethasone in the hydrogel (Supporting Information). This confirms that the original dose of dexamethasone in the hydrogel delivery system was within experimental error quantitatively released during the time studied.

Figure 7 shows that the simulated dexamethasone concentration in the vitreous compartment throughout the experiment was maintained well above therapeutic levels (>1 nM, or 0.393 ng/mL, gray dashed line),^8,9^ making the formulation a good candidate for the treatment of chronic inflammatory retinal diseases. Based on the model, vitreal drug concentrations would be maintained above therapeutic levels for 320 days after a single injection (Supporting Information). The model is based on the assumption that the hydrogel of released polymer conjugates is not eliminated from the eye. Since this assumption is likely not true, the accuracy of the model beyond 57 days is uncertain, and the duration of action should be verified experimentally.

The release of dexamethasone from the hydrogel is due to hydrolysis of the ester in the linker that connects the drug with the hydrogel matrix (see Scheme 1).^16,22^ Previously, we measured dexamethasone release from PNADEX-CA hydrogel in vitro (PBS pH 7.4, 37 °C) for over 400 days with a *k*_r_ of 0.0017 day^–1^, corresponding to a half-life of 408 days.^16^ However, the hydrolytic half-life of the free mDEX monomer under the same condition was only ∼6 days.^16^ This slower release of dexamethasone from the hydrogel compared to the free monomer was ascribed to the lower water activity in the hydrophobic domains of the hydrogel.^49,50^ In the present study, we determined the in vivo release rate of dexamethasone from the PNADEX-CA hydrogel (0.042 day^–1^), being approximately 25 times faster than the release rate in vitro (0.0017 day^–1^).^16^ In the in vitro experiments, hydrogels were formed inside a glass vial, resulting in a well-defined cylindrical shape, with only one surface exposed to the release medium. In contrast, in vivo release occurred in three dimensions. During the in vivo experiments, hydrogels were injected into the vitreous space after cross-linking. Likely, after injection into the eye, the preformed hydrogel formulation had a larger and irregular surface area due to shear-induced temporary softening of the material and water uptake.

Although dexamethasone release in vitro is governed by ester hydrolysis only, in vivo, this process may be accelerated by enzymatic degradation of the gel. Hydrolysis of the ester bonds in the aqueous environment of the vitreous is probably catalyzed by carboxylesterases.^51,52^ This leads to the formation of smaller gel fragments and better enzyme access, further speeding up dexamethasone release. In addition to the ester bonds present in the linker that connects dexamethasone to the polymer, the ester groups present in the polymer backbone itself, flanking the PEG block, may be cleaved,^53^ allowing degradation of the hydrogel. Overall, the combination of hydrolysis by water and enzymatic activity in vivo likely resulted in significantly faster dexamethasone release compared to that in vitro.

In vivo, the unlabeled PNADEX-CA hydrogel could not be visualized using a fundus camera 2 weeks after injection, and the fluorescently labeled PNADEX-BDP-CA hydrogel could be visualized up to 3 weeks in the rat vitreous. Although the use of a fluorescence label in the PNADEX-BDP-CA hydrogel aided in the visualization of the polymeric material in the rat vitreous, it could also result in a lower the cross-link density of the network since the NHS groups of the polymer were used to attach the label. However, it was shown that only one out of 20 polymer chains (see Supporting Information) contained a fluorescent label, which makes it unlikely that the labeling affected cross-linking efficiency. As we tested the labeled and unlabeled hydrogel delivery systems in different animal models, with different injection volumes, direct comparison of the degradation rates of the labeled and unlabeled hydrogels is difficult.

## 4 Conclusions

The present study shows that the PNADEX-CA hydrogel system released dexamethasone in rabbit eyes for at least 2 months, while the simulation suggests that therapeutic levels of dexamethasone in the vitreous may be maintained even longer. This makes the hydrogel a promising delivery system for the treatment of chronic ocular inflammatory diseases. Even though mild trauma-induced adverse effects were observed in half of the rat eyes, likely related to the injection procedure, histological analysis of the rat retina, however, did not show any morphological changes between the treated and untreated control eyes. Moreover, no adverse effects caused by the hydrogels were observed in the eyes of the rabbits. The rat and rabbit studies showed that hydrogel degradation and clearance, as well as dexamethasone release, are faster in vivo than in vitro. More studies on degradation are needed to fully assess the pharmacokinetics, safety, and efficacy of the long-term use of the formulation.

## Supplementary Material



## Notes

Additional experimental details on the characterizations of the synthesized polymers, labeling and the injectable hydrogels; additional fundus images; and dexamethasone concentrations over time (PDF)
