# Supplementary material for: Intravitreal Sustained Release of Dexamethasone from a Self-Healing Injectable Hydrogel: An In Vivo Safety and Release Study
Source: Mol Pharm. 2025 Sep 30;22(11):6920–31. doi: 10.1021/acs.molpharmaceut.5c00872 (PMC12587444; doi:10.1021/acs.molpharmaceut.5c00872)
Supplement: Supplementary file 1 [file mp5c00872_si_001.pdf]

Supplementary information:

Intravitreal Sustained Release of Dexamethasone from a Self-Healing Injectable Hydrogel: An in Vivo Safety and Release Study

Ada Annala, Amir Sadeghi, Elisa Toropainen, Annika Valtari, Jooseppi Puranen, Jussi J. Paterno, Lea Pirskanen, Kati-Sisko Vellonen, Wim E. Hennink, Marika Ruponen, Tina Vermonden, Arto Urtti.

Table S1: Tissue processing procedure

Solvent	Time
Tap water	10 minutes
80% ethanol	30 minutes
94% ethanol	30 minutes
94% ethanol	30 minutes
99% ethanol	45 minutes
99% ethanol	30 minutes
99% ethanol	30 minutes
Xylene	20 minutes
Xylene	20 minutes
Liquid paraffin	1 hour
Liquid paraffin	1 to 24 hours

Table S2: H&E staining protocol

Reagent	Time
Xylene	2 x 5 minutes
100 % ethanol	2 x 2 minutes
94 % ethanol	2 x 2 minutes
Rinsing with distilled water	20 seconds
Harris hematoxylin	5 minutes
Rinsing with tap water	5 minutes

1 % HCl in 70% ethanol	4-5 seconds
Rinsing with tap water	10 minutes
1 % eosin	30 seconds
94 % ethanol	2 x 2 minutes
100 % ethanol	2 x 2 minutes
Xylene	2 x 5 minutes

Xylene (BDH Prolabo, VWR Chemicals, France), and 100% and 94% ethanol (Altia Oyj, Finland) were purchased from manufacturers. Delafield hematoxylin, 1% HCl in 70% ethanol and 1% eosin were prepared in the University of Eastern Finland. Prior staining, the 1% eosin was filtered and 1 ml of glacial acetic acid (BDH Prolabo, VWR Chemicals, France) was added to 100ml 1% eosin. Glass slides were covered with Depex mounting medium (provider) and samples were let to dry for 18 to 30 hours. Excess stain was removed from the plates before microscopy imaging.

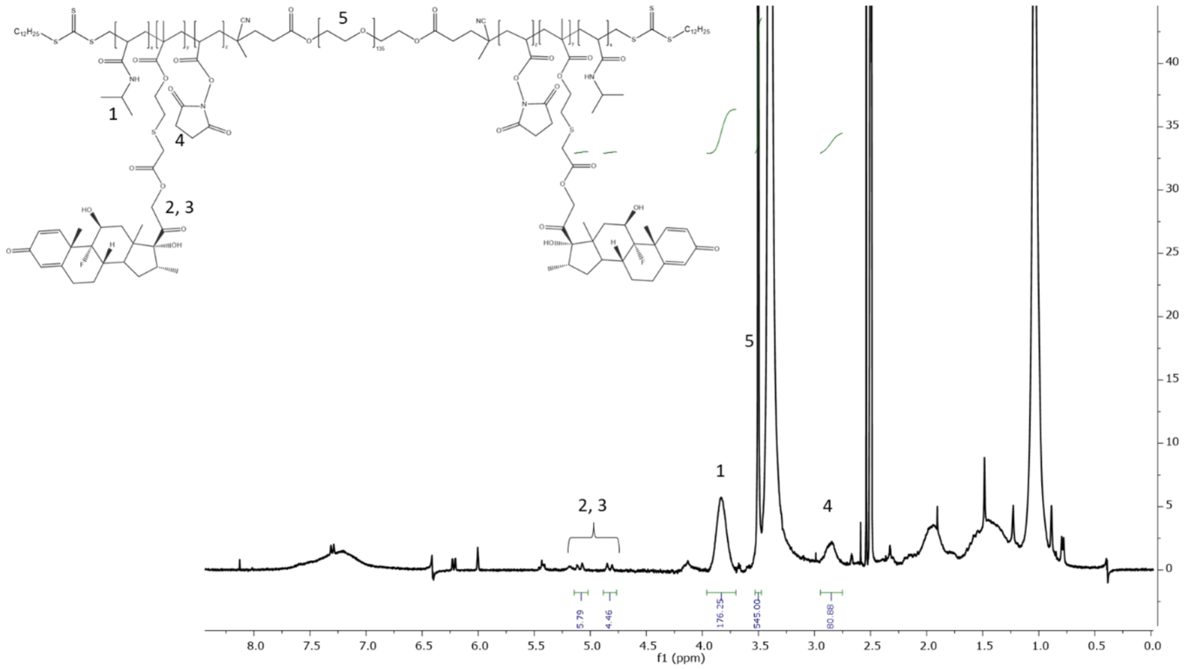

Figure S1: ^{1}H NMR spectrum of PNADEX in DMSO-d6. Residual solvent peaks are observed at 2.50 ppm (DMSO) and 3.33 ppm (H_2O).

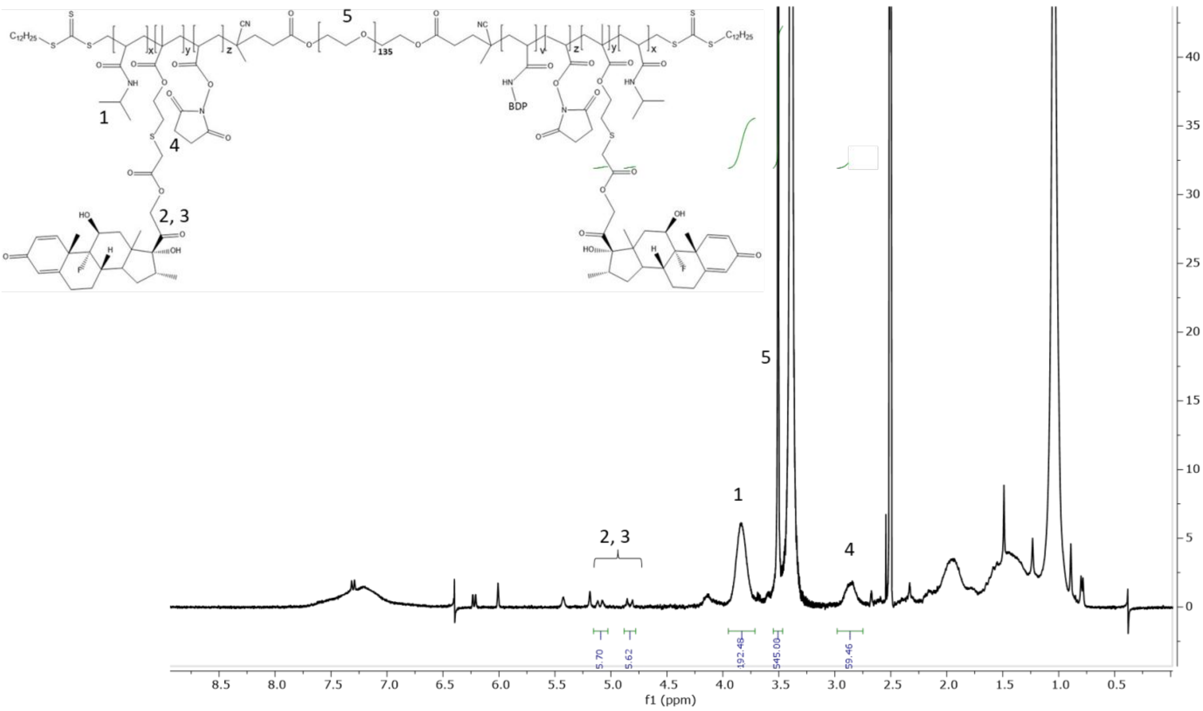

Figure S2: ^{1}H NMR spectrum of PNADEX-BDP in DMSO-d6. Residual solvent peaks are observed at 2.50 ppm (DMSO) and 3.33 ppm (H_2O).

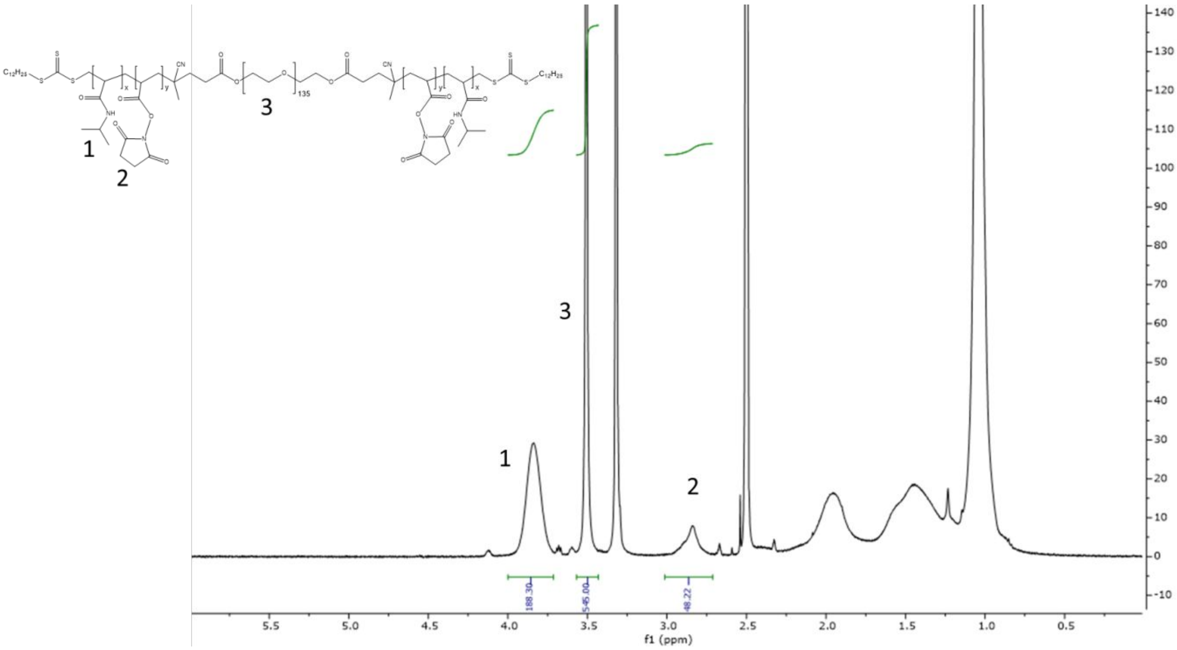

Figure S3: ^{1}H NMR spectrum of PNA in DMSO-d6. Residual solvent peaks are observed at 2.50 ppm (DMSO) and 3.33 ppm (H_2O).

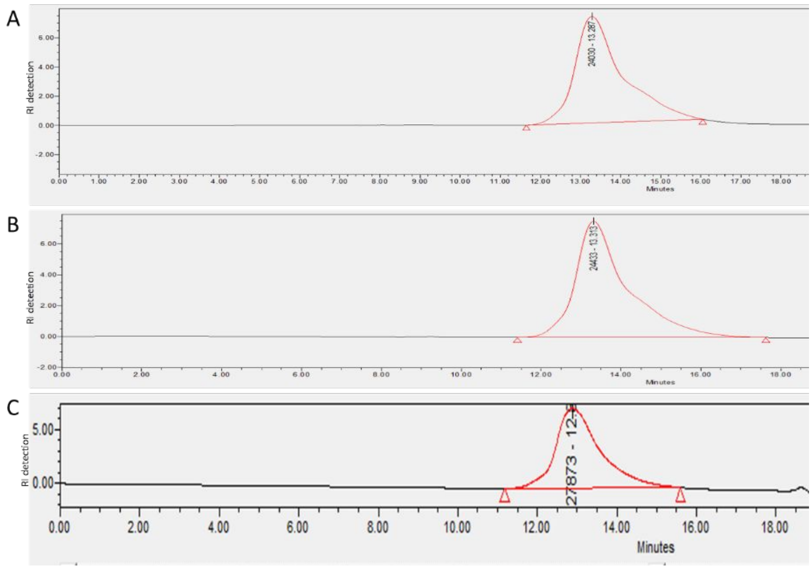

Figure S4: GPC chromatograms of A) PNADEX B) PNADEX-BDP and C) PNA polymers

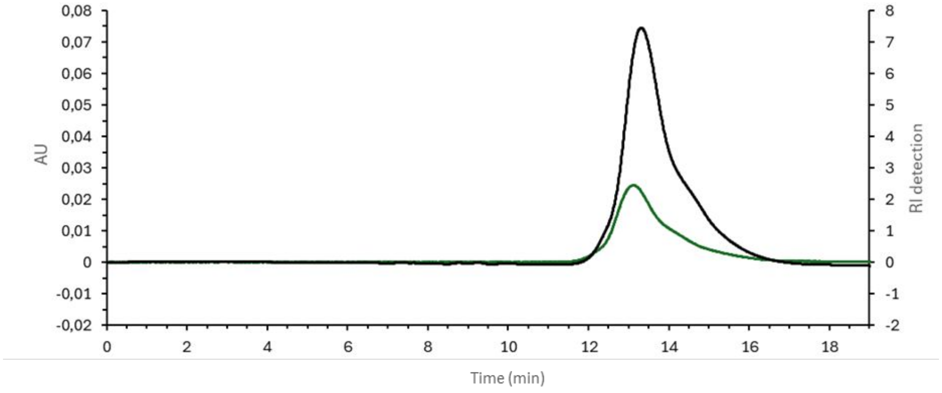

Figure S5: GPC chromatogram of fluorescently labelled PNADEX-BDP using RI detection (black line) and UV detection at 500 nm (green line). Single peak overlapping with RI signal could be observed for the sample using UV detector, indicating absence of free unconjugated dye in the polymer. (RI= refractive index; AU= absorbance unit.)

Determination of labelling degree (LD) of PNADEX-BDP

The absorption spectra of PNADEX-BDP dissolved in DI water at concentrations 0, 1.25, 2.5, and 5 mg/mL were recorded (Figure S6A) and absorbance at λ =503 nm is plotted against the PNADEX-BDP concentration (Figure S6B). Linear curve fitting was used to obtain the extinction coefficient for the polymer (slope=0.205 mL mg^{-1} cm^{-1}). The molar extinction coefficient for PNADEX-BDP ($\varepsilon_{PNADEX-BDP}$) was calculated from the slope, using the molecular weight of 35.3 kDa for the polymer; 0.205 mL mg^{-1} cm^{-1} * 353000 g/mol = 7237 mL $mmol^{-1}$ cm^{-1}. The LD of PNADEX-BDP was then calculated using the molar extinction coefficient of BDP dye (ε_{BDP})

(92000 mL mmol^{-1} cm^{-1}, provided by Lumiprobe) according to the equation below. The labelling degree of 0.079 which means one out of ~13 polymer chains carry a dye molecule

$$LD_{BDP} = \frac{\varepsilon_{PNADEX-BDP}}{\varepsilon_{BDP}} = \frac{7237}{92000} = 0.079$$

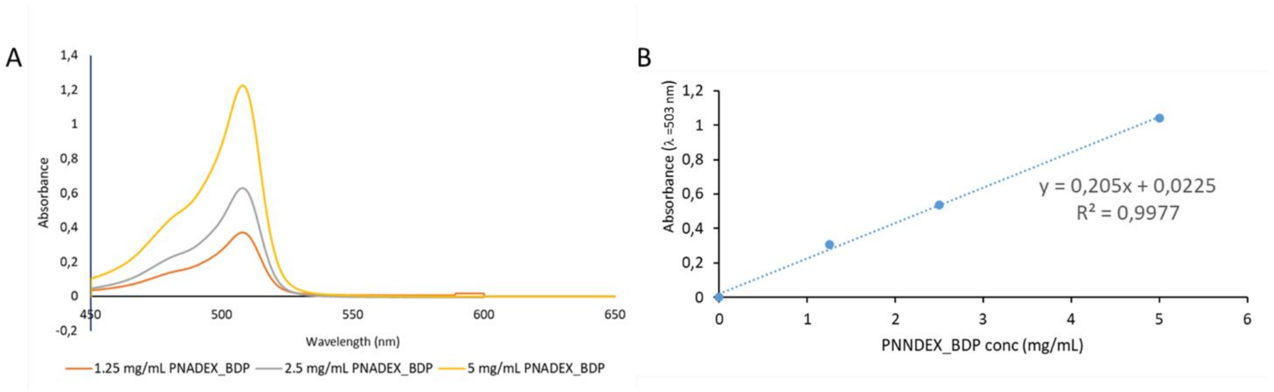

Figure S6: (A) Absorbances of PNADEX-BDP aqueous solutions at concentrations 1.25, 2.5 and 5 mg/ml. (B) Linear curve fitting to obtain the extinction coefficient of 0.205 mL mg^{-1} cm^{-1}for PNADEX-BDP polymer (λ=503 nm).

Calculation of the coupling efficiency of BDP dye to the polymer:

71 mg of PNADEX polymer was reacted with 3.5 mg dye. Assuming 100 % conjugation efficiency, 5 mg of the labelled polymer then contains $\frac{3.5\ mg}{71\ mg + 3.5\ mg} \cdot 5\ mg =$ 0.23 mg dye.

When 5 mg of this polymer is dissolved in 1 ml buffer (corresponding with 5 mg/mL), the resulting concentration of dye is 0.23 g/L /427 g/mol (MW of the dye) = $0.54 \cdot 10^{-3}$ mol/L.

The absorbance of the solution at 502 nm in a 1 cm cuvette is then ($A = \epsilon \cdot conc\ \cdot cm$) with ε provided by manufacturer is 92*10^3 L.Mol$^{-1}\cdot$cm^{-1}): $A = 0.54 \cdot 10^{-3} \cdot 92 \cdot 10^3 \cdot 1 = 49.6$.

The observed absorbance at 5 mg/mL PNADEX-BDP was 1.05. This means that the coupling efficiency is 1.05/49.6 = 2.1%.

Considerations regarding hydrogel formation:

During the crosslinking reaction, the NHS esters react with the amine crosslinker to form amide bonds. Unwanted chemical hydrolysis of the NHS esters may also occur, although Shah et al. (Journal of Controlled Release, 45 (1997) 95–101) reported that such hydrolysis from a copolymer of N-isopropylacrylamide (NIPAm) and N-acryloxysuccinimide (NAS) proceeded very slowly. At pH 8 and 37 °C, only about 10% of the NHS esters were hydrolyzed after 10 hours, whereas in our previous study, hydrogel formation was complete within 3 hours at the same temperature and at an even lower pH of 7.4. Thus, although chemical hydrolysis cannot be entirely avoided, it is likely a minor factor.

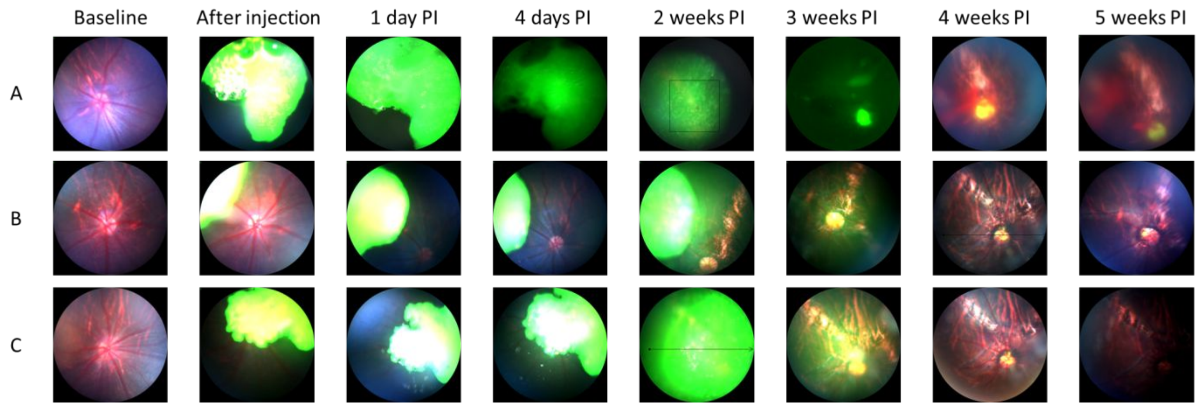

Figure S7: Fundus images of three different rat eyes (A-C) after 5 µL injection of 10 wt% PNADEX-BDP-CA hydrogel (PI= post injection). Images were taken at the baseline, immediately after injection, 1 and 4 days after injection and weekly between 2- and 5-weeks post injection.

In Vivo release of dexamethasone

The dexamethasone concentrations were quantified from the rabbit aqueous humor samples with LC-MS/MS, and the results are presented in Table S3.

Table S3: Dexamethasone concentrations in aqueous humor samples at different timepoints

Timepoint (days)	Eye ID	Concentration dexamethasone (ng/ml)	Average (ng/ml)	SD
3	1	16,8	18,4	6,0
	2	14,1		
	3	27,6		
	4	22,7		
	5	11,0		
7	1	23,1	22,9	5,0
	2	17,6		
	3	26,8		
	4	29,7		
	5	17,1		
15	1	14,5	15,0	6,4
	2	27,4		
	3	11,5		
	4	11,3		
	5	10,3		
22	1	9,6	9,2	0,7
	2	9,3		
	3	10,2		
	4	8,8		
	5	8,2		

29	1	8,9	7,2	1,0
	2	7,3		
	3	6,4		
	4	6,2		
	5	7,1		
38	1	4,0	4,3	0,4
	2	5,0		
	3	4,2		
	4	3,9		
	5	4,5		
44	1	6,7	4,0	1,4
	2	3,0		
	3	2,9		
	4	3,5		
	5	3,9		
51	1	4,0	1,7	0,9
	2	1,7		
	3	1,7		
	4	1,6		
	5	1,8		
57	1	2,0	1,4	0,3
	2	1,2		
	3	1,3		
	4	1,2		
	5	1,3		

Comparison of $AUC_{0\text{-}57d}$ to the initial dose of dexamethasone

To estimate the remaining dose of dexamethasone inside the hydrogel at day 57, which was the last sampling timepoint in our experiment, the following approach was used.

Using the model presented in Figure 1, the calculated $AUC_{0\text{-}57d}$ for intravitreal dexamethasone was 43.8 µg.day/mL

Since

$\text{Clearance (CL)} = k_{vit} \cdot V_d \text{ (volume of distribution)} = 5.55\ \text{day}^{-1} \cdot 1.5\ \text{mL} = 8.325\ \text{mL/day}$

It is further known that $\text{Dose} = \text{AUC} \cdot \text{CL}$

The AUC for 402 µg of dexamethasone ($AUC_{0\text{-}inf}$) is

$$402\ \mu\text{g}/8.325\ \text{mL} \cdot \text{day}^{-1} = 48.3\ \mu\text{g} \cdot \text{day} \cdot \text{mL}^{-1}$$

Thus $AUC_{0\text{-}57d}$ / $AUC_{0\text{-}inf}$

$$\text{AUC}_{0-57\text{d}}/\text{AUC}_{0-\text{inf}} = \frac{43.8}{48.3} * 100\% = 91\%$$

Therefore, at day 57, the delivery system has released 91 % of the total dose, with 9% of dose (corresponding to 36 µg of dexamethasone) still remaining inside the hydrogel.

Calculation of the duration of therapeutic dexamethasone concentrations in the vitreous

The time reach the minimal therapeutic dexamethasone concentration in the vitreous after injection of the hydrogel formulation is calculated as follows assuming first order elimination kinetics:

$C_t = C_0 e^{-kt}$ or $ln\, C_t = ln\, C_0 - kt$

thus

$$t = \frac{1}{k} ln\left(\frac{C_t}{C_0}\right)$$

Where:

C_t= concentration at time t
C_0= concentration at time 0 = 402000 ng/1.5 mL= 268000 ng/mL
x= minimal therapeutic concentration, 0.39 ng/mL [8,9]
t= time
k = reaction rate constant of dexamethasone release from the hydrogel, 0.042 day^{-1}

thus: t = ln(268000 ng/mL /0.39 ng/mL)/0.042 day^{-1}= 320 days
